# Does Adjusting for Causal Intermediate Confounders Resolve the Perinatal Crossover Paradox?

**DOI:** 10.1097/EDE.0000000000001848

**Published:** 2025-02-25

**Authors:** Wen Wei Loh, Cande V. Ananth

**Affiliations:** From the ^a^Department of Methodology and Statistics, Faculty of Health, Medicine and Life Sciences (FHML), Maastricht University, Maastricht, The Netherlands; b Division of Epidemiology and Biostatistics, Department of Obstetrics, Gynecology, and Reproductive Sciences, Rutgers Robert Wood Johnson Medical School, New Brunswick, NJ; c Cardiovascular Institute of New Jersey, Rutgers Robert Wood Johnson Medical School, New Brunswick, NJ; d Department of Medicine, Rutgers Robert Wood Johnson Medical School, New Brunswick, NJ; e Department of Biostatistics and Epidemiology, Rutgers School of Public Health, Piscataway, NJ; f Environmental and Occupational Health Sciences Institute (EOHSI), Rutgers Robert Wood Johnson Medical School, New Brunswick, NJ.

**Keywords:** Causal mediation analysis, Exposure-dependent or exposure-induced confounding, Interventional indirect effects, Multiple mediators, Perinatal mortality, Preeclampsia, Preterm birth or delivery

## Abstract

**Background:**

Mediation analyses of the preeclampsia–perinatal outcome association through preterm birth (PTB) have produced paradoxical findings. For example, preeclamptic births at preterm gestations show a lower risk of adverse outcomes than normotensive births. These results have been explained by unmeasured baseline confounding between PTB and outcomes, with PTB as the sole mediator. However, other intermediate variables, such as placental abruption, small for gestational age (SGA) births, and chorioamnionitis, are confounders yet are excluded because they occur after preeclampsia.

**Methods:**

Using data from the Consortium on Safe Labor (2002–2008; N=203,990), we utilized interventional indirect effects to examine whether adjusting for causal intermediates mitigates confounding bias to resolve the perinatal paradox. We compared two approaches to handle intermediate confounding by abruption, SGA, and chorioamnionitis when PTB is the focal mediator: as exposure-induced confounders or as multiple mediators. We developed bias formulas to assess unmeasured confounding for interventional effects.

**Results:**

When PTB was the sole mediator, the estimated protective direct effect of preeclampsia (risk ratio = 0.60; 95% confidence interval = 0.52, 0.71) was in line with previous paradoxical findings. The estimated protective effect persisted even after adjusting for intermediate confounders. Sensitivity analyses suggested an unmeasured confounder must strongly influence the outcome to resolve the paradox.

**Conclusion:**

Adjusting for causal intermediates such as abruption, SGA, and chorioamnionitis is inadequate to eliminate unmeasured PTB–perinatal mortality confounding. The paradox of preeclampsia’s protective direct effect on mortality remains unresolved. Sensitivity analyses to unmeasured confounding are effective in bolstering conclusions from causal mediation analyses and should be more widely applied.

Mediation analyses with preterm birth (PTB) mediating the causal association between preeclampsia and perinatal outcomes (such as perinatal mortality or cerebral palsy) have resulted in paradoxical findings. These studies suggest that, at preterm gestations, the risks of an adverse outcome among preeclamptic births were either similar^1^ or lower^2,3^ than those among normotensive births. These counterintuitive findings about the direct effect of preeclampsia have been explained by collider-stratification bias^4,5^ resulting from unmeasured baseline confounding between PTB and the outcome.^6–12^

This perinatal paradox has long been recognized in other contexts: at preterm gestations, perinatal mortality rates are lower among women who smoke than among nonsmokers^13–17^; twins compared with singletons^18,19^; Black compared with White persons^20–22^; hypertensive compared with normotensive persons^23–26^; obese persons compared with persons with normal body mass index,^27^ to name a few. Interestingly, mortality rates among the “exposed” group exhibit a crossover phenomenon with increased mortality rates at term gestations. The important covariate that is common to all such paradoxes is gestational age.

In virtually all studies that have produced this paradox, PTB was the sole intermediate variable. Other variables causally intermediate between preeclampsia and perinatal mortality, for instance, such as placental abruption, small for gestational age (SGA) birth, and chorioamnionitis are also risk factors for PTB (our focal mediator)^28^ and predict perinatal mortality.^29^ On the one hand, they are confounders of the mediator–outcome association and must be adjusted for to minimize unmeasured confounding bias. On the other hand, they occur temporally after a preeclampsia diagnosis (exposure) and must be excluded when using routine techniques to control for baseline confounding.

We investigate whether adjusting for these causal intermediate confounders can resolve the paradox that the effect of preeclampsia on perinatal mortality has a protective direct effect with PTB. We focus on interventional effects^30–32^ that permit accounting for intermediate confounders when decomposing the total effect of preeclampsia on perinatal mortality into an indirect effect through PTB and a direct effect via causal pathways other than PTB.^33,34^ Because intermediate confounders occur after preeclampsia, they cannot be considered baseline or pre-exposure confounders.^35^ Therefore, we compare two competing causal mediation analytic approaches that can handle these intermediate confounders alongside our focal mediator: as exposure-induced confounders^30^ or as multiple mediators.^36,37^ Both approaches allow estimating the direct and indirect effects through PTB as the focal mediator but differ in whether to include other intermediate variables as exposure-induced confounders or as mediators. We hope our presentation will help researchers make informed decisions depending on their specific research context by illustrating the causal effect estimands and assumptions side-by-side.

Notwithstanding adjustments for intermediate confounders, interventional (in)direct effects remain susceptible to bias due to uncontrolled confounding. Therefore, to assess the sensitivity of mediation analysis results to putative unmeasured confounding, we develop bias formulas that can counteract unmeasured confounding in the presence of intermediate confounders. The bias correction formulas extend the existing method^38^ for natural (in)direct or controlled direct effects for a single mediator, that is, without any other causally intermediate variables, to interventional (in)direct effects allowing for either exposure-induced confounders or multiple mediators. While the developed bias formulas generally allow for a set of unmeasured confounders (e.g., previous miscarriages, urinary tract infection, cocaine and other substance use, or congenital anomalies, among others), to ease the presentation and interpretation of the results, we assume a single binary unmeasured confounder as a simplifying assumption in line with extant approaches.^11,38^ In general, sensitivity analyses to the unmeasured mediator–outcome confounding can unveil new perspectives into the absence of specific causal pathways (such as the direct effect) that would otherwise have been present and are an effective tool to bolster conclusions from causal mediation analyses that should be applied broadly in practice.

## METHODS

We conducted a secondary analysis of data from the Consortium on Safe Labor (CSL) project.^39,40^ The CSL was designed as a retrospective cohort study and included data abstracted from electronic medical records between 2002 and 2008 across 19 hospitals in 12 clinical centers in the United States. Because these data are deidentified and publicly available from the National Institute of Child Health and Human Development’s Data and Specimen Hub repository, no institutional review board approval was sought. We used preeclampsia as the primary exposure and perinatal mortality as the outcome. We defined perinatal mortality as a stillbirth at ≥23 weeks in gestation or neonatal deaths within the first month of life. We examined PTB as the focal mediator. We defined PTB as <37 completed weeks, with gestational age in the CSL based on the best obstetrical estimate. Baseline covariates, jointly denoted by C, included study site, maternal age (years), parity, marital status, race/ethnicity, insurance type, smoking status, alcohol use during pregnancy, prepregnancy body mass index, height, pregestational diabetes, gestational diabetes mellitus, and calendar year of delivery.

We will focus on three variables causally intervening between preeclampsia and perinatal mortality as putative intermediate confounders of the PTB–perinatal mortality association: placental abruption, fetal growth restriction or SGA birth, and chorioamnionitis. In the CSL, abruption diagnosis was based on clinical findings abstracted from the labor-delivery records or discharge diagnosis codes (based on the International Classification of Disease, 9th revision). SGA was defined as sex-specific birthweight less than the 10th percentile at every gestation week, derived using data from the current study. Chorioamnionitis is an ascending infection that originates in the lower genitourinary tract and migrates to the amniotic cavity. The infection is often the result of the rupture of the chorioamniotic membranes and is diagnosed at delivery. Although preeclampsia may not be directly associated with chorioamnionitis, many preeclamptic pregnancies undergo labor induction^28^ and are often preceded by an artificial or spontaneous membrane rupture. Long labors, particularly among primiparous patients, allow the possibility of ascending chorioamnionitis. Chorioamnionitis, in turn, is associated with increased mortality risk.^41^ Depending on the chosen approach, these variables can act as exposure-induced confounders or multiple mediators, as described in the following section.

### Interventional (In)Direct Effect Causal Estimands

#### Definitions and interpretations

**FIGURE 1.**
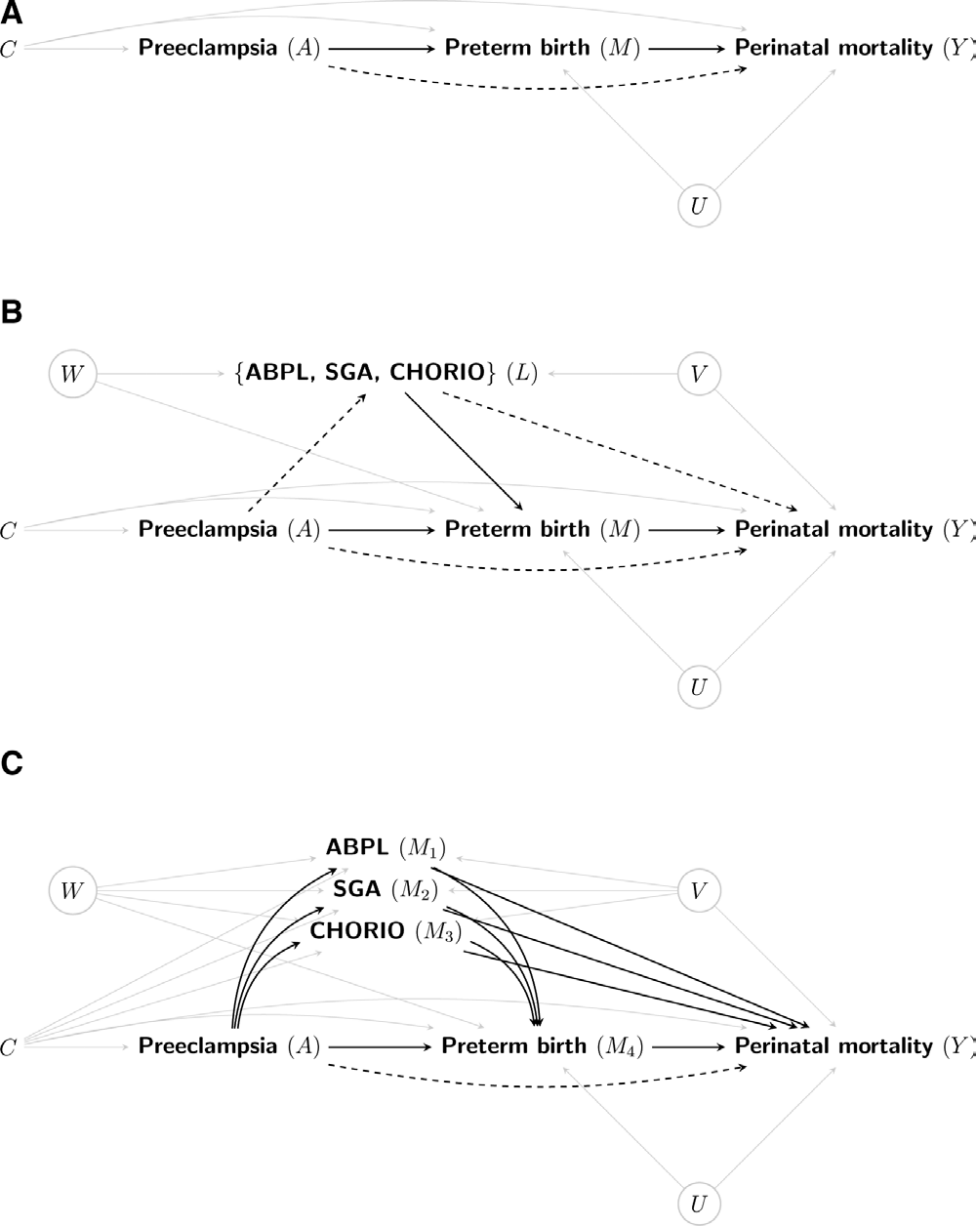
Causal diagrams depicting the pathways through preterm birth as the focal mediator of the preeclampsia–perinatal mortality association, when preterm birth is either (A) the sole intermediate variable; (B) influenced by exposure-induced confounders; or (C) alongside other mediators. ABPL, placental abruption; CHORIO, chorioamnionitis. The dashed paths contribute to the direct effect of preeclampsia on perinatal mortality bypassing (all) the mediator(s). Round nodes denote unmeasured variables. Arrows emanating from confounders are drawn in gray for visual clarity.

We use the interventional indirect effects framework for causal mediation analysis.^30–32^ Our choice is motivated by the ability to compare (almost) the same causal estimand for the indirect effect through PTB across three different approaches: as a single mediator (ignoring all other intermediate variables), with exposure-induced confounders, or alongside multiple mediators. The causal diagrams representing each approach are displayed in Figure 1.

We define the interventional (in)direct causal effect estimands using potential outcomes with counterfactual mediators.^42^ We first present the single mediator approach under which all other intermediate variables are ignored. Let $Y\left(a_{0},\tilde{M}\left(a_{1}\right)\right)$ denote the potential outcome Y that would have been observed if, possibly contrary to fact, the exposure A was set to $a_{0}$, and the mediator M was set to a random draw from its counterfactual distribution under $A=a_{1}$, conditional on the measured baseline covariates C. The stochastic nature of the latter is indicated notationally by the tilde (∼) symbol in $\tilde{M}\left(a_{1}\right)$, and we omit the dependence on C for notational simplicity. We focus on causal effects defined on the risk ratio (RR) scale for a binary exposure A. The interventional direct effect is:

$$\begin{array}{l} RR_{DE}\left(1\right)=\frac{E\left[Y\left(1,\tilde{M}\left(1\right)\right)\right]}{E\left[Y\left(0,\tilde{M}\left(1\right)\right)\right]}. \end{array}$$ (1)

This direct effect compares the perinatal mortality risk between preeclamptic and non–preeclamptic births if, in both cases, PTB was set to its counterfactual distribution under preeclampsia ($a_{1}=1$). Throughout, the number(s) in the brackets of a direct or indirect effect indicate the fixed hypothetical exposure level(s) for other causal pathways. For example, for the direct effect $RR_{DE}\left(1\right)$, the number 1 refers to the hypothetical exposure level for the mediator’s counterfactual distribution. It describes the causal effect of preeclampsia along other (possibly unknown) pathways bypassing PTB. We chose this specific definition because our interest is in the “total” direct effect^42^ of preeclampsia under an elevated risk of PTB (under preeclampsia); in contrast, the controlled direct effect of preeclampsia in previous analyses^2,3^ stratified on actual PTBs.

The interventional indirect effect is:

$$\begin{array}{l} RR_{IE}\left(0\right)=\frac{E\left[Y\left(0,\tilde{M}\left(1\right)\right)\right]}{E\left[Y\left(0,\tilde{M}\left(0\right)\right)\right]}. \end{array}$$ (2)

In our application, this “pure” indirect effect^42^ assesses the mortality risk if we were unable versus able to induce PTB under its counterfactual distribution without preeclampsia among births without preeclampsia ($a_{0}=0$). This answers the clinically relevant question: among women without preeclampsia, to what extent does PTB lead to poorer infant and maternal outcomes than term delivery? Conceptually, this is the causal effect of preeclampsia along all causal pathways leading to PTB, then directly to perinatal mortality.^36^ The RR for the total effect is the product of the indirect and direct effects, that is,

$$\begin{array}{l} RR_{TE}=RR_{DE}\left(1\right)\times RR_{IE}\left(0\right). \end{array}$$ (3)

We now consider the approach that includes other causal intermediate variables, such as placental abruption, SGA, and chorioamnionitis, as joint exposure-induced confounders, henceforth denoted by L. Under this approach, the causal effect estimands in (1) and (2) and their decomposition in (3) are the same.^30^ However, the interpretations of the interventional effects are different, as we explain using Figure 1B. The path-specific effects along causal paths intersecting L but bypassing M, such as A→L→Y, are subsumed under the direct effect. The path-specific effects via L and intersecting M, such as A→L→M→Y, are subsumed under the indirect effect. Hence, maintaining the same estimands, either ignoring or including exposure-induced confounders, allows focusing on whether adjusting for intermediate confounders can eliminate unmeasured mediator–outcome confounding while minimizing differences in the definitions, identification assumptions, and estimation procedures.

We now turn to the approach using multiple mediators. Instead of designating the other intermediate variables (placental abruption, SGA, and chorioamnionitis) as exposure-induced confounders, we will consider them simultaneous mediators alongside our focal mediator (PTB); see Figure 1C. There are two benefits of adopting the latter approach over the former. First, the total effect can be decomposed into separate mediator-specific indirect effects to gain insights into different causal pathways linking preeclampsia and perinatal mortality.^34,43^ In contrast, the causal effects transmitted through intermediate variables designated as exposure-induced confounders are combined with the direct effect.^36^ Second, interventional effects for multiple mediators require less stringent assumptions about the causal structure among the intermediate variables,^36^ albeit at the expense of more strict assumptions about the mediator–outcome relations. We elaborate on these assumptions later in the context of identification.

Following our application with four causal intermediate variables (placental abruption, SGA, chorioamnionitis, and PTB), we index the mediators by $M_{j},j=1,2,3,4$. However, we emphasize that the indices are arbitrary and need not reflect any causal sequence or order. We will use $Y\left(a_{0},{\tilde{M}}_{1}\left(a_{1}\right),{\tilde{M}}_{2}\left(a_{2}\right),{\tilde{M}}_{3}\left(a_{3}\right),{\tilde{M}}_{4}\left(a_{4}\right)\right)$ to denote the potential outcome that would have been observed if, possibly contrary to fact, $A=a_{0}$, and the counterfactual mediators $M_{j},j=1,2,3,4$, were set to random draws from their counterfactual distributions under $A=a_{j}$, respectively.

The interventional direct effect is:


$$\begin{array}{l} RR_{DE}\left(1,1,1,1\right)=\frac{E\left[Y\left(1,{\tilde{M}}_{1}\left(1\right),{\tilde{M}}_{2}\left(1\right),{\tilde{M}}_{3}\left(1\right),{\tilde{M}}_{4}\left(1\right)\right)\right]}{E\left[Y\left(0,{\tilde{M}}_{1}\left(1\right),{\tilde{M}}_{2}\left(1\right),{\tilde{M}}_{3}\left(1\right),{\tilde{M}}_{4}\left(1\right)\right)\right]}. \end{array}$$ (4)

In our application, this interventional direct effect compares the perinatal mortality risk between preeclamptic and non–preeclamptic births if, in both cases, all four mediators $M_{j},j=1,2,3,4$, were respectively set to their counterfactual distributions under a preeclampsia diagnosis ($a_{j}=1$). Hence, the numbers in $RR_{DE}\left(1,1,1,1\right)$ refer to the hypothetical exposure levels for the mediators’ counterfactual distribution. We chose this definition in (4) because its interpretation is closest to the “total” direct effect for a single mediator in (1), in the sense that all the mediators are drawn from their counterfactual distribution under preeclampsia.

We index PTB as $M_{4}$ and the other causal intermediates by $\left\{M_{1},M_{2},M_{3}\right\}$ (instead of L). Define the interventional indirect effect through $M_{4}$ as:


$$\begin{array}{l} RR_{IE_{4}}\left(0,0,0,0\right)=\frac{E\left[Y\left(0,{\tilde{M}}_{1}\left(0\right),{\tilde{M}}_{2}\left(0\right),{\tilde{M}}_{3}\left(0\right),{\tilde{M}}_{4}\left(1\right)\right)\right]}{E\left[Y\left(0,{\tilde{M}}_{1}\left(0\right),{\tilde{M}}_{2}\left(0\right),{\tilde{M}}_{3}\left(0\right),{\tilde{M}}_{4}\left(0\right)\right)\right]}. \end{array}$$ (5)

In our application, this interventional indirect effect through PTB provides an assessment of the mortality risk if we were unable versus able to induce PTB under its counterfactual distribution without preeclampsia, among non–preeclamptic births ($a_{0}=0$) and with the counterfactual distribution of all other mediators without preeclampsia ($a_{1}=a_{2}=a_{3}=0$). Hence, the numbers in $RR_{IE_{4}}\left(0,0,0,0\right)$ refer to the hypothetical exposure levels for other effects bypassing the focal mediator (e.g., the direct effect and indirect effects via other mediators). We chose this definition in (5) because its interpretation is closest to the “pure” indirect effect for a single mediator in (2), in the sense that all hypothetical exposure levels excluding the focal mediator ($M_{4}$), especially the other mediators ($M_{1},M_{2},M_{3}$), are under non–preeclampsia. Similar to the previous settings, we can interpret the indirect effect through $M_{4}$ as the combined effect of A along all (possibly unknown) causal paths, including via other mediators, leading to $M_{4}$, then directly to Y.^36^

The indirect effects for the remaining mediators can be similarly defined so that the total effect is decomposed as:


$$\begin{array}{l} RR_{TE}=RR_{DE}\left(1,1,1,1\right)\times RR_{IE_{1}}\left(0,1,1,1\right)\times RR_{IE_{2}}\left(0,0,1,1\right)\;\times RR_{IE_{3}}\left(0,0,0,1\right)\times RR_{IE_{4}}\left(0,0,0,0\right). \end{array}$$ (6)

#### Identification

The causal effect estimands defined earlier can be identified under certain assumptions of no unmeasured confounding. For the single mediator approach, the average potential outcome can be identified as^36^:


$$\begin{array}{l} E\left[Y\left(a_{0},\tilde{M}\left(a_{1}\right)\right)\right]=\underset{c}{\sum }\;\underset{m}{\sum }\;E\left[Y|A=a_{0},M=m,C=c\right] \end{array}$$

$$\begin{array}{l} \;\;\;\;\times P\left(M=m|A=a_{1},C=c\right)P\left(C=c\right), \end{array}$$ (7)

when there is no unmeasured confounding between A and either M or Y, conditional on C, and no unmeasured confounding between M and Y, conditional on {A,C}.

When there are exposure-induced confounders L, the average potential outcome can be identified as^30^:


$$\begin{array}{l} \begin{array}{lll} E\left[Y\left(a_{0},\tilde{M}\left(a_{1}\right)\right)\right] & =\underset{c}{\sum }\;\underset{l,m}{\sum }\;E\left[Y|A=a_{0},L=l,M=m,C=c\right]\; & \times \;P\left(L=l|A=a_{0},C=c\right)\; \end{array} \end{array}$$

$$\begin{array}{l} \;\;\;\;\times \;P\left(M=m|A=a_{1},C=c\right)P\left(C=c\right), \end{array}$$ (8)

when there is no unmeasured confounding between A and either M or Y, conditional on C, and no unmeasured confounding between M and Y, conditional on {A,C,L}. Hence, omitting L from adjustment can induce unmeasured confounding.

When there are multiple mediators, the average potential outcome is identified as^36^:


$$\begin{array}{l} E\left[Y\left(a_{0},{\tilde{M}}_{1}\left(a_{1}\right),{\tilde{M}}_{2}\left(a_{2}\right),{\tilde{M}}_{3}\left(a_{3}\right),{\tilde{M}}_{4}\left(a_{4}\right)\right)\right] \end{array}$$

$$\begin{array}{lll} \;\;\;\;=\underset{c}{\sum }\;\underset{m_{1},m_{2},m_{3},m_{4}}{\sum }\; & E[Y|A=a_{0},M_{1}=m_{1},M_{2}=m_{2},\; & M_{3}=m_{3},M_{4}=m_{4},C=c] \end{array}$$

$$\begin{array}{l} \;\;\;\;\times \underset{j=1}{\overset{4}{\prod }}\;P\left(M_{j}=m_{j}|A=a_{j},C=c\right)P\left(C=c\right), \end{array}$$ (9)

when there is no unmeasured confounding between A and the mediators $M_{j},j=1,2,3,4$, or Y, conditional on C, and no unmeasured confounding between the mediators jointly and Y, conditional on {A,C}. In particular, note that the latter assumption (under weak union^44^) implies no unmeasured confounding between the focal mediator $\left(M_{4}\right)$ and outcome (Y), conditional on $\left\{A,M_{1},M_{2},M_{3},C\right\}$. Therefore, omitting any of $\left\{M_{1},M_{2},M_{3}\right\}$ from adjustments can introduce unmeasured confounding.

We briefly compare the identification assumptions for the exposure-induced confounding and multiple-mediator approaches using the causal diagrams in Figure 1B and C, respectively. The former permits unmeasured confounding between the intermediate confounders designated as L (i.e., excluding the focal mediator M) and Y, such as V in Figure 1B, but is predicated on L causally preceding M with no unmeasured confounding between them, such as W in Figure 1B because W would induce “butterfly” bias^45^ between M and Y when adjusting for L. In contrast, the latter precludes unmeasured confounding of all intermediate variables and the outcome, such as V in Figure 1C, but permits unknown causal ordering and unmeasured confounding among the intermediate variables, such as W in Figure 1C. Both require no unmeasured confounding between the focal mediator and outcome, conditional on {A,C}. A more detailed exposition of the identification assumptions under each approach is presented in Web Appendix; http://links.lww.com/EDE/C226.

#### Missing data

There were missing values for a few baseline covariates in C. To account for the missingness, we applied single stochastic imputations with chained equations for each covariate predicted by the others in C using random forests,^46^ assuming data were missing at random. In each bootstrap sample, this imputation was used to complete the dataset before estimating the interventional effects. There were no missing data for the exposure (preeclampsia), intermediate confounders (placental abruption, SGA, and chorioamnionitis), mediator (PTB), or outcome (perinatal mortality).

#### Sensitivity analysis to unmeasured confounding

Notwithstanding adjustment for intermediate confounders, there remains the possibility of unmeasured baseline mediator–outcome confounding,^47,48^ such as by in Figure 1. We conducted a sensitivity analysis using bias formulas.^38^ For each approach earlier, we developed a closed-form expression of the bias factor (on the RR scale) for the interventional (in)direct effects allowing for intermediate confounders. Because the mathematical derivations of the formulas are relatively lengthy, for example, requiring additional sensitivity parameters encoding the associations between the focal mediator and intermediate confounders, interested readers are referred to the Web Appendix; http://links.lww.com/EDE/C226. The derived bias formulas for each mediation approach, under simplifying assumptions about the unmeasured confounder and its associations with the observed variables, are described in Table 1. The bias factor (under fixed values of the sensitivity parameters) is then applied to the effect estimate to counteract the bias due to unmeasured confounding. Systematically varying the sensitivity parameter values permits judging the extent to which the conclusions are altered by unmeasured confounding.

**TABLE 1.** Bias Factors for Interventional (In)Direct Effects due to Unmeasured Mediator–Outcome Confounding Under Each Approach Assuming a Single Binary Unmeasured Pre-Exposure Confounder

Approach	Direct Effect	Indirect Effect
Single mediator	$\frac{1+\left(\gamma -1\right)\pi_{1}}{1+\left(\gamma -1\right)\pi_{0}}$	$\frac{1+\left(\gamma -1\right)\pi_{0}}{1+\left(\gamma -1\right)\pi_{1}}$
Exposure-induced confounders	$\frac{1+\left(\gamma^{2}-1\right)\pi_{1}}{1+\left(\gamma^{2}-1\right)\pi_{0}}\times \frac{1+\left(\gamma -1\right)\pi_{0}}{1+\left(\gamma -1\right)\pi_{1}}$	$\frac{1+\left(\gamma -1\right)\pi_{0}}{1+\left(\gamma -1\right)\pi_{1}}$
Multiple mediators	$\frac{1+\left(\gamma^{2}-1\right)\pi_{1}}{1+\left(\gamma^{2}-1\right)\pi_{0}}\times \frac{1+\left(\gamma -1\right)\pi_{0}}{1+\left(\gamma -1\right)\pi_{1}}$	$\frac{1+\left(\gamma -1\right)\pi_{0}}{1+\left(\gamma -1\right)\pi_{1}}$

γ_,_ effect of binary unmeasured confounder on the outcome (on the risk ratio scale); $\pi_{a}$_,_ prevalence of confounder within each stratum defined by A=a.

## RESULTS

**FIGURE 2.**
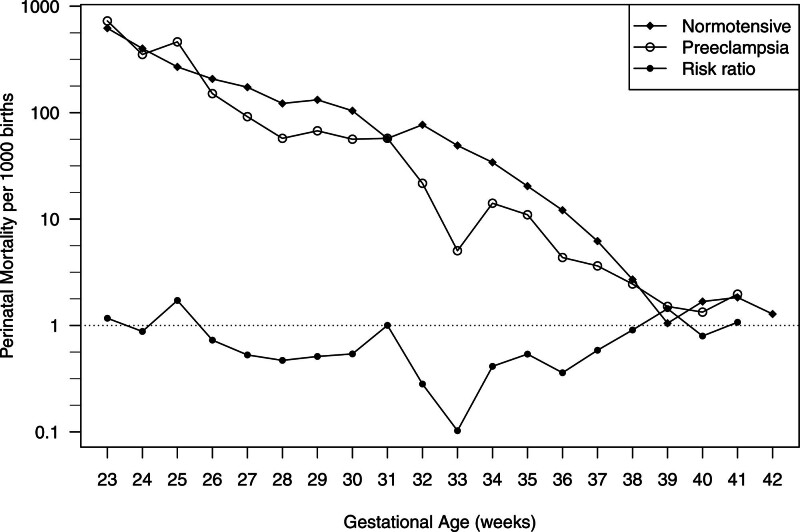
Gestational age-specific risk of perinatal mortality (per 1000 births) among preeclamptic and normotensive births, and risk ratio of mortality between preeclamptic and normotensive births from Consortium on Safe Labor Project, 2002–2008. At 42 weeks, the risk among preeclamptic births was exactly zero.

Of the 203,990 singleton births, were preeclamptic births. The distributions of maternal characteristics by preeclampsia are shown in Table 2. Compared with patients with non–preeclamptic births, the proportion of persons who were primiparous, of single marital status, non-Hispanic Blacks, having private insurance type, being obese, pregestational diabetes, or gestational diabetes was increased among those with preeclamptic births. Among preeclamptic births, were delivered at preterm gestations; of these preterm deliveries, and were clinician-initiated and spontaneous preterm deliveries, respectively. Among non–preeclamptic births, these proportions were for PTB, with clinician-initiated and spontaneous PTB, respectively. Gestational age-specific perinatal mortality rates were almost uniformly lower for preeclamptic births than normotensive births (Figure 2).

**TABLE 2.** Distribution of Risk Factors in Relation to Preeclampsia From Consortium on Safe Labor Project, 2002–2008

Risk Factors	Total Births		Preeclampsia		No Preeclampsia	
	n	%	n	%	n	%
Number of births	203,990	100.0	12,002	100.0	191,988	100.0
Maternal age (years)						
<18	6,988	3.4	537	4.5	6,451	3.4
18–19	12,658	6.2	960	8.0	11,698	6.1
20–24	51,032	25.0	2,965	24.7	48,067	25.0
25–34	102,284	50.1	5,383	44.9	96,901	50.5
35-39	24,396	12.0	1,578	13.1	22,818	11.9
≥40	6,333	3.1	558	4.6	5,775	3.0
Missing	299	0.1	21	0.2	278	0.1
Parity						
Primiparous	89,030	43.6	6,927	57.7	82,103	42.8
Parity ≥2, no previous stillbirth	90,016	44.1	3,804	31.7	86,212	44.9
Parity ≥2, previous stillbirth	432	0.2	48	0.4	384	0.2
Missing	24,512	12.0	1,223	10.2	23,289	12.1
Marital status						
Married	118,921	58.3	5,986	49.9	112,935	58.8
Single	78,723	38.6	5,667	47.2	73,056	38.1
Missing	6,346	3.1	349	2.9	5,997	3.1
Race/ethnicity						
Non-Hispanic White	100,077	49.1	4,827	40.2	95,250	49.6
Non-Hispanic Black	45,808	22.5	3,877	32.3	41,931	21.8
Hispanic	35,730	17.5	2,067	17.2	33,663	17.5
Other	13,393	6.6	731	6.1	12,662	6.6
Missing	8,982	4.4	500	4.2	8,482	4.4
Insurance type						
Government	113,332	55.6	6,295	52.4	107,037	55.8
Private	66,041	32.4	4,396	36.6	61,645	32.1
Other	2,763	1.4	200	1.7	2,563	1.3
Missing	21,854	10.7	1,111	9.3	20,743	10.8
Smoking status						
Nonsmoker	190,232	93.3	11,285	94.0	178,947	93.2
Smoker	13,758	6.7	717	6.0	13,041	6.8
Alcohol use						
No	200,201	98.1	11,803	98.3	188,398	98.1
Yes	3,789	1.9	199	1.7	3,590	1.9
Prepregnancy BMI (kg/m^2^)						
Underweight (<18.5)	7,453	3.7	226	1.9	7,227	3.8
Normal (18.5–24.9)	73,201	35.9	2,794	23.3	70,407	36.7
Overweight (25.0–29.9)	30,594	15.0	1,890	15.7	28,704	15.0
Obese (30.0–34.9)	14,312	7.0	1,198	10.0	13,114	6.8
Obese-plus (≥35.0)	11,006	5.4	1,242	10.3	9,764	5.1
Missing	67,424	33.1	4,652	38.8	62,772	32.7
Height (cm)						
Quartile 1 (<158)	37,196	18.2	2,431	20.3	34,765	18.1
Quartile 2 (158–162)	46,339	22.7	2,651	22.1	43,688	22.8
Quartile 3 (163–168)	42,253	20.7	2,375	19.8	39,878	20.8
Quartile 4 (>168)	43,660	21.4	2,331	19.4	41,329	21.5
Missing	34,542	16.9	2,214	18.4	32,328	16.8
Pregestational diabetes						
Absent	201,014	98.5	11,462	95.5	189,552	98.7
Present	2,976	1.5	540	4.5	2,436	1.3
Gestational diabetes						
Absent	193,442	94.8	10,972	91.4	182,470	95.0
Present	10,548	5.2	1,030	8.6	9,518	5.0
Year of delivery						
2002–2004	30,279	14.8	2,115	17.6	28,164	14.7
2005–2008	173,711	85.2	9,887	82.4	163,824	85.3
Induction of labor						
No	131,696	64.6	5,577	46.5	126,119	65.7
Yes	72,294	35.4	6,425	53.5	65,869	34.3
Placental abruption						
No	200,664	98.4	11,633	96.9	189,031	98.5
Yes	3,326	1.6	369	3.1	2,957	1.5
Small for gestational age						
No	180,961	88.7	9,640	80.3	171,321	89.2
Yes	23,029	11.3	2,362	19.7	20,667	10.8
Chorioamnionitis						
Absent	197,292	96.7	11,659	97.1	185,633	96.7
Present	6,698	3.3	343	2.9	6,355	3.3
Preterm birth						
Term delivery ≥37 weeks	179,972	88.2	7,396	61.6	172,576	89.9
Spontaneous preterm <37 weeks	16,051	7.9	1,893	15.8	14,158	7.4
Clinician-initiated preterm <37 weeks	7,967	3.9	2,713	22.6	5,254	2.7

BMI, body mass index.

Under all three approaches, we estimated interventional effects for the preeclampsia–perinatal mortality association, with PTB as the focal mediator. In the Appendix, we briefly describe the estimation procedure for each approach. The results are shown in Table 3. We make three observations. First, controlling for only baseline covariates and ignoring putative exposure-induced confounders resulted in a protective direct effect (RR = 0.60; 95% confidence interval [CI] = 0.52, 0.71), in line with existing counterintuitive findings. Second, including placental abruption, SGA, and chorioamnionitis as exposure-induced confounders attenuated the direct effect slightly toward the null . This suggested that adjusting for these intermediate confounders was inadequate to eliminate the unmeasured PTB–perinatal mortality confounding. Third, considering placental abruption, SGA, chorioamnionitis, and PTB as multiple mediators amplified the direct effect away from the null . This suggested that unmeasured confounding of mediators and the outcome could potentially exacerbate biases in the direct effect. Adjustments for the intermediate confounders placental abruption, SGA, and chorioamnionitis do not resolve the paradox of a protective direct effect of preeclampsia unmediated via PTB.

**TABLE 3.** Interventional Effects of Preeclampsia on Perinatal Mortality Through Preterm Birth Using Each Approach: Consortium on Safe Labor Project, 2002–2008

Approach	Direct Effect		Indirect Effect		Total Effect	
	RR	95% CI	RR	95% CI	RR	95% CI
Single mediator (preterm birth)	0.60	(0.52, 0.71)	2.59	(2.46, 2.72)	1.56	(1.34, 1.82)
Exposure-induced confounders	0.64	(0.55, 0.76)	2.41	(2.28, 2.53)	1.54	(1.33, 1.80)
Multiple mediators	0.53	(0.46, 0.64)	2.42	(2.29, 2.55)	1.49	(1.29, 1.77)

The causal intermediate covariates between preeclampsia and perinatal mortality considered as either exposure-induced confounders or multiple mediators were placental abruption, SGA births, and chorioamnionitis, with preterm birth as the focal mediator.

**FIGURE 3.**
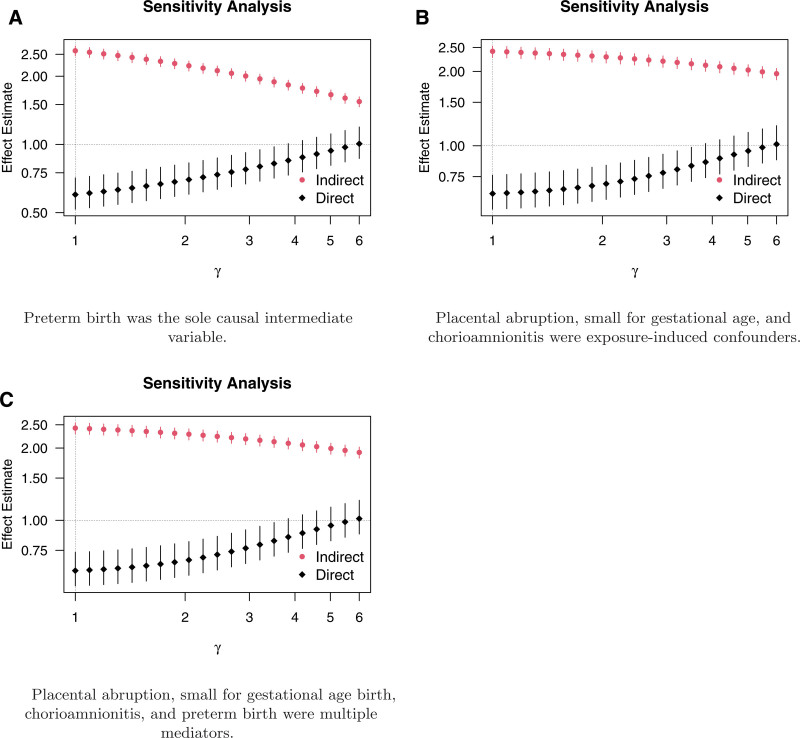
Sensitivity analysis to unmeasured preterm birth–perinatal mortality baseline confounding under the three considered approaches. Estimates of the interventional indirect effect through preterm birth and the direct effect bypassing the mediator(s) are plotted. Vertical bars indicate the 95% confidence intervals. The sensitivity parameter γ encodes the effect of a binary unmeasured confounder on the outcome, conditional on all other observed variables, on the risk ratio scale. A value of γ=1 corresponds to the observed estimates (i.e., assuming no unmeasured confounding). A, Preterm birth was the sole causal intermediate variable. B, Placental abruption, small for gestational age, and chorioamnionitis were exposure-induced confounders. C, Placental abruption, small for gestational age birth, chorioamnionitis, and preterm birth were multiple mediators.

We undertook a sensitivity analysis of unmeasured confounding for the three approaches. We varied the unmeasured confounder’s effect on the outcome on the RR scale, conditional on all other observed variables, as parametrized by γ in our bias formulas. All other sensitivity parameters were fixed at values based on the observed data; see details in Web Appendix; http://links.lww.com/EDE/C226. The results are plotted in Figure 3. Across all three approaches, as γ increased, the direct effect became greater than one, indicating a detrimental effect of preeclampsia on mortality unmediated via PTB. This conclusion aligned with previous sensitivity analyses.^11^

## DISCUSSION

Preeclampsia confers a strong perinatal mortality risk to the infant.^29,49^ As a consequence, these pregnancies are subject to obstetrical interventions at preterm gestations^50^ in an effort to prevent stillbirth and neonatal mortality and, to some extent, improve maternal health. Early delivery, in and of itself, confers increased mortality risk,^48^ and in the setting of preeclampsia, the mortality risk is substantially higher. The clinically important question is what the increased risk of perinatal mortality due to preeclampsia is and to what extent PTB shapes the increased risk. Attempts to address this result in a paradox: the direct effect of preeclampsia on mortality is protective, whereas an effect mediated through PTB shows a higher risk. A similar paradox has also been identified based on birthweight, with low birthweight (<2500 g) infants born of pregnancies with a high-risk exposure (e.g., smoking mothers) having improved perinatal survival than infants with high birthweights.^14,51^ In this article, we considered four causal intermediates of preeclampsia and perinatal mortality: abruption, SGA births, chorioamnionitis, and PTB.

The recognition of this perinatal paradox arguably dates back six decades when Yerushalmy^17^ documented that infant mortality rates at preterm gestations were lower among infants born to smoking than nonsmoking mothers.^15^ This paradox related to smoking extended to the current context of preeclampsia. While preeclampsia confers a higher average risk of perinatal mortality, when restricting the analysis to preterm deliveries only, preeclampsia might appear to counterintuitively lower,^2,3^ or have no effect,^1^ on the risk of death among babies born early. Because these results have been attributed to collider bias due to unmeasured confounding between PTB and the outcome, adjusting for causal intermediates as confounders may pull the estimate closer to the null toward resolving this paradox. However, adjusting for abruption, SGA births, and chorioamnionitis had a negligible estimated impact on the direct effect estimate (from 0.60 to 0.64) and was inadequate to eliminate the unmeasured PTB–perinatal mortality confounding. We conjecture that adjusting for the three exposure-induced confounders did not substantively change the conclusions because none were especially strong confounders of the PTB–perinatal mortality association. We found no indirect effects via these other putative mediators (up to sampling error), assuming that sequential ignorability was met when considering them as multiple mediators. A similar conclusion that SGA was not strongly associated with perinatal mortality and the association of placental abruption and mortality was attenuated after adjusting for PTB was previously found.^37^ Our sensitivity analyses suggested the unmeasured confounder must strongly influence the outcome to resolve the paradox of a protective direct effect of preeclampsia unmediated via PTB.

Several theories have been proposed to explain the paradox. It has been suggested that maternal stress due to preeclampsia provides a survival advantage to fetuses at preterm gestations, afforded by advanced fetal lung maturation.^52^ However, this hypothesis has also been challenged.^53^ From an epidemiologic design perspective, it has been argued that selection bias^54^ may have led to the paradox. Virtually all studies in perinatal epidemiology begin the observation window at 20 weeks (or later) in gestation. Unhealthy pregnancies, in general, which result in spontaneous losses before 20 weeks, are removed from the “risk set.” This left truncation can be a concern because pregnancies that end due to miscarriage or spontaneous abortion before preeclampsia is clinically diagnosed are systematically excluded from a study, thereby inducing a form of selection bias^55^ (a “healthy survivor” bias), resulting in the paradox. However, because the CSL study analyzed in this article uses a birth cohort approach, exploring missing data methods that account for informative left truncation in suitable study designs^55^ is beyond the scope of this article and deferred to future work.

Another form of selection bias arises when associations are stratified on the causal intermediate–PTB.^14,48^ This article focused on comparing two different methods to adjust for intermediator confounders toward understanding the mechanisms driving the paradoxical associations of preeclampsia with perinatal mortality. For simplicity, we operationalized definitions of SGA as sex-specific birthweight less than the 10th percentile at every gestational age and PTB as obstetrical interventions at preterm gestations <37 completed weeks. Nonetheless, using such broad discretized categories may introduce some residual confounding. We acknowledge that, in principle, a severe form of SGA (e.g., less than the 5th or 3rd percentile) or PTB (e.g., at <34 or <32 weeks) could be considered. Examining the impact of such definitions is beyond the scope of our article and is an important avenue for future work.

The fetuses-at-risk approach has been proposed as another methodology that overcomes the paradox.^56^ This method essentially avoids collider stratification by including fetuses still in the uterus in the population at risk of death.^57^ In other words, the fetuses-at-risk derivation of stillbirth rates, for example, is estimated based on the denominator of the total population “at risk” at a given gestational age (which includes both delivered and ongoing pregnancies at the given gestational age). Although estimation of relative effects (relative risk or hazard ratio) based on the fetuses-at-risk conceptualization has been proposed,^57,58^ methods for causal decomposition for that setting remain to be developed.

The gestational age at delivery marks the end of a pregnancy, and PTB is defined for both stillbirth and live birth using the same definition (<37 weeks). Preeclampsia is one of three chief indications for PTB (the other two being placental abruption and fetal growth restriction^28^). Preeclampsia is a strong risk factor for stillbirth, and pregnancies that end at preterm gestation (irrespective of preeclampsia) carry an increased risk of stillbirth. While stillbirth may be considered a competing event for PTB, stillbirth (like preeclampsia) is an indication of an obstetrical intervention at preterm gestations. Given the complex associations, it is arguably premature to classify all stillbirths as a competing event for PTB. Moreover, PTB can be classified into spontaneous or medically indicated preterm deliveries.^43^

Other attempts to resolve the paradox include analyzing mortality risks associated with a given exposure based on predicted risks of the causal intermediate^11^ (Basso and Wilcox^59^ estimated that about half of the mortality of singleton preterm babies was due to the pathologies that cause early delivery) but preclude estimating the indirect effect, especially in the presence of exposure-induced confounding. We put forth that sensitivity analyses for uncontrolled mediator–outcome confounding using bias correction formulas^38^ is a straightforward tool that should be routine in all mediation analyses. While simplifying assumptions are often imposed to ease the practical use of these bias formulas and their results, sensitivity analysis techniques can still be informative even if precise values of the sensitivity parameters cannot be ascertained in practice.^11^

Adjusting for intermediate confounders strengthens the justification for no unmeasured mediator–outcome confounding. We compared two mediation analysis approaches accounting for causal intermediates as potential confounders between the exposure and outcome. We focused on interventional indirect effects^43^ because they can be identified in the presence of causal intermediate confounders under weaker assumptions than natural or path-specific effects, which require assuming a correct causal structure and no unmeasured confounding among mediators,^60,61^ and cross-world independencies.^62^ Another practical challenge is the causal consistency assumption when conceptualizing a preeclamptic patient’s counterfactual outcome had that specific individual been hypothetically normotensive. However, therein lies a strength of the interventional effects approach adopted in our article: counterfactual mediators can be interpreted as population-level stochastic, dynamic interventions instead of individual-level deterministic interventions used in natural indirect effects.^63^ Therefore, the mean potential outcome (e.g., perinatal mortality) for preeclamptic patients can be interpreted as being averaged over the counterfactual distribution of PTB under a reduced likelihood of developing preeclampsia, for example, through hospital-level policy recommendations or expected management of preeclamptic pregnancies. Such interventions are more clearly defined and clinically meaningful.

Because the mediator PTB was relatively rare among non–preeclamptic births (e.g., only 2.7% were clinician-initiated), there was a potential for positivity violations whereby PTB could be perfectly predicted. However, there were no extreme estimates in the fitted multinomial logistic regression models (see the results in Web Appendix; http://links.lww.com/EDE/C226) that would suggest complete separation. On the absolute scale, overall mortality rates are higher among preeclamptic pregnancies than normotensive pregnancies. However, the paradox emerges when rates are stratified on a collider (gestational age), with the effects on the RR scale showing protective effects of preterm preeclampsia on mortality. This unexpected finding runs the counterintuitive interpretation that it is empirically justifiable to deliver preterm preeclamptic pregnancies insofar as reducing perinatal mortality.

## CONCLUSION

Studies of the direct effect of preeclampsia (unmediated via PTB) have produced paradoxical findings of a protective association between preeclampsia and mortality among PTBs. These results have been attributed to collider bias due to unmeasured confounding between PTB and the outcome. In this article, we examined whether adjusting for placental abruption and SGA birth as intermediate confounders mitigated the impact of unmeasured confounding. We focused on the interventional direct effect that bypasses PTB and the interventional indirect effect through PTB. We compared two approaches: treating intermediate confounders as exposure-induced confounders or as multiple mediators alongside PTB. Using data from the CSL project, our results demonstrated that adjusting for these intermediate confounders remains inadequate to eliminate the unmeasured PTB–perinatal mortality confounding. Our sensitivity analyses suggested the unmeasured confounder must strongly influence the outcome to resolve the paradox of a protective direct effect of preeclampsia unmediated via PTB. In general, sensitivity analyses to unmeasured mediator–outcome confounding should be widely used in mediation analysis.

## ACKNOWLEDGMENTS

The authors thank the Editor, Sunni L. Mumford, for constructive suggestions and valuable advice in guiding the manuscript through the review process. The authors are grateful to Justin S. Brandt, K. S. Joseph, Rachel Lee, Todd Rosen, Anthony M. Vintzileos, and two anonymous referees for their insightful comments that helped improve the manuscript.

The data included in this study were obtained from the Consortium on Safe Labor, supported by the Intramural Research Program of the Eunice Kennedy Shriver National Institute of Child Health and Human Development, National Institutes of Health (Contract No. HHSN267200603425C). Institutions involved in the Consortium on Safe Labor include, in alphabetical order: Baystate Medical Center, Springfield, MA; Cedars-Sinai Medical Center Burnes Allen Research Center, Los Angeles, CA; Christiana Care Health System, Newark, DE; Georgetown University Hospital, MedStar Health, Washington, DC; Indiana University Clarian Health, Indianapolis, IN; Intermountain Healthcare and the University of Utah, Salt Lake City, Utah; Maimonides Medical Center, Brooklyn, NY; MetroHealth Medical Center, Cleveland, OH.; Summa Health System, Akron City Hospital, Akron, OH; The EMMES Corporation, Rockville MD (Data Coordinating Center); University of Illinois at Chicago, Chicago, IL; University of Miami, Miami, FL; and University of Texas Health Science Center at Houston, Houston, TX.

## APPENDIX

### Estimation

For the single mediator approach, we fitted two regression models: (i) a binary logistic model for outcome mortality (PND) given pre-eclampsia (PE), PTB, and all baseline covariates (C); and (ii) a multinomial logistic model for the mediator PTB given PE and C. Using these two fitted models, for each individual, we randomly drew a value of PTB from its counterfactual distribution using (ii) under the hypothetical pre-eclampsia scenario of $A=a_{1}$, conditional on observed C. Each random draw of the counterfactual PTB (under $A=a_{1}$) was then entered, with observed C and pre-eclampsia set to $A=a_{0}$, into the fitted outcome model in (i) to predict the potential outcome (7). This approach to randomly drawing counterfactual mediators and predicting the potential outcomes was repeated 200 times for each individual. The average predicted potential outcomes across all random draws and individuals yielded an estimator of the average potential outcome. Such Monte Carlo-based counterfactual simulations are commonly employed for estimating causal effects.^36,64^

For the exposure-induced confounding approach, we fitted five regression models: (i$\;\;{\;}^{\prime }$) A binary logistic model for PND given PE, PTB, all exposure-induced confounders (placental abruption [ABPL], SGA, and chorioamnionitis [CHORIO]), and C; (ii$\;\;{\;}^{\prime }$) A multinomial logistic model for PTB given PE, ABPL, SGA, CHORIO, and C; (iii$\;\;{\;}^{\prime }$) A binary logistic model for ABPL given PE and C; and (iv$\;\;{\;}^{\prime }$) A binary logistic model for SGA given PE, ABPL, and C; and (v$\;\;{\;}^{\prime }$) A binary logistic model for CHORIO given PE, ABPL, SGA, and C. Models (iii$\;\;{\;}^{\prime }$), (iv$\;\;{\;}^{\prime }$), and (v$\;\;{\;}^{\prime }$) were used to factorize the joint probability of the exposure-induced confounders, given PE and C, into a product of iterative conditional probabilities.

Using the fitted models for each individual, we first randomly drew values of the exposure-induced confounders (ABPL, SGA, and CHORIO) sequentially from their counterfactual distributions using (iii$\;\;{\;}^{\prime }$), (iv$\;\;{\;}^{\prime }$), and (v$\;\;{\;}^{\prime }$) under the hypothetical pre-eclampsia scenario of $A=a_{1}$, conditional on observed C. We then randomly drew a value of PTB from its counterfactual distribution using (ii$\;\;{\;}^{\prime }$) under the same hypothetical pre-eclampsia scenario of $A=a_{1}$, conditional on the random draws of ABPL, SGA, and CHORIO also under $A=a_{1}$, and observed C. Next, we randomly drew values of ABPL, SGA, and CHORIO sequentially from their counterfactual distributions using (iii$\;\;{\;}^{\prime }$), (iv$\;\;{\;}^{\prime }$), and (v$\;\;{\;}^{\prime }$) under $A=a_{0}$, conditional on observed C. These random draws were entered, with the random draw of the counterfactual PTB (under $A=a_{1}$), observed C, and pre-eclampsia set to $A=a_{0}$, into the fitted outcome model in (i$\;\;{\;}^{\prime }$) to predict the potential outcome (8). This approach of randomly drawing counterfactual mediators and predicting the potential outcomes was repeated 200 times for each individual. The average predicted potential outcomes across all random draws and individuals yielded an estimator of the average potential outcome. Our procedure follows the parametric mediational g-formula for time-varying confounders and mediators^65,66^ for a single mediator timepoint.

For the multiple-mediator approach, we also fitted five regression models: (i${\;\;\;}^{*}$) A binary logistic model for PND given PE, PTB, ABPL, SGA, and C; and (ii${\;\;\;}^{*}$) A mediator model for each of ABPL ($M_{1}$), SGA ($M_{2}$), CHORIO ($M_{3}$), and PTB ($M_{4}$) given PE and C. For the mediator models in (ii${\;\;\;}^{*}$), binary logistic models were used for ABPL, SGA, and CHORIO; and a multinomial logistic model was used for PTB. Each mediator model in (ii${\;\;\;}^{*}$) encoded the marginal effect of PE, unconditional on the other mediators, on that mediator given C.^36^ The outcome models in (i$\;\;{\;}^{\prime }$) and (i${\;\;\;}^{*}$) were identical, as were the mediator models for PTB in (ii) and (ii${\;\;\;}^{*}$). Using the fitted models, for each individual, we randomly drew a value of each mediator $M_{j},j=1,2,3,4$, from its counterfactual distribution using (ii${\;\;\;}^{*}$) under its specific hypothetical pre-eclampsia scenario of $A=a_{j}$, conditional on observed C. Each random draw of the counterfactual mediator (under $A=a_{j}$) was then entered, with observed C and pre-eclampsia set to $A=a_{0}$, into the fitted outcome model in (i${\;\;\;}^{*}$) to predict the potential outcome in (9). This method of random drawing counterfactual mediators and predicting the potential outcomes was repeated 200 times for each individual. The average predicted potential outcomes, across all random draws and individuals, yielded an estimator of the average potential outcome.^36^

Under each approach, the interventional direct and indirect effects were calculated using the average potential outcomes corresponding to the specific hypothetical exposure levels under comparison. We used a nonparametric bootstrap approach with 1999 samples drawn with replacement to estimate 95% CIs. Statistical analysis was performed using SAS (version 9.4; SAS Institute, Cary, NC) and R (version 4.2.3; R Core Team, Vienna, Austria). The R scripts to implement the mediation analyses can be found on GitHub (https://github.com/wwloh/perinatal-multiple-mediation).
